# Convenience without disclosure: a formative research study of a proposed integrated methadone and antiretroviral therapy service delivery model in Dar es Salaam, Tanzania

**DOI:** 10.1186/s13722-017-0089-6

**Published:** 2017-10-18

**Authors:** Alexis Cooke, Haneefa Saleem, Dorothy Mushi, Jessie Mbwambo, Saria Hassan, Barrot H. Lambdin

**Affiliations:** 1 Fielding School of Public Health Department of Community Health Sciences, University of California, Los Angeles, Los Angeles, CA USA; 2 Department of International Health, Johns Hopkins Bloomberg School of Public Health, 615 North Wolfe Street, E5033, Baltimore, MD 21205 USA; 3 Department of Psychiatry, Muhimbili University of Health and Allied Sciences, P.O. Box 65293, Dar es Salaam, Tanzania; 4 Behavioral Health and Criminal Justice Research Division, RTI International, 351 California Street, Suite 500, San Francisco, CA 94104 USA

## Abstract

**Background:**

Though timely initiation of antiretroviral therapy (ART) is a vital component of effective HIV prevention, care and treatment, people who inject drugs are less likely to receive ART than their non-drug using counterparts. In an effort to increase access to ART for people who inject drugs, we examined perceived benefits, challenges, and recommendations for implementing an integrated methadone and ART service delivery model at an opioid treatment program (OTP) clinic in Dar es Salaam, Tanzania.

**Methods:**

We conducted in-depth interviews with 12 providers and 20 HIV-positive patients at the Muhimbili National Hospital OTP clinic in early 2015. We used thematic content analysis to examine patient and provider perspectives of an integrated model.

**Results:**

Respondents perceived that offering on-site CD4 testing and HIV clinical management at the OTP clinic would improve the timeliness and efficiency of the ART eligibility process, make HIV clinical care more convenient, mitigate stigma and discrimination in HIV care and treatment settings, and improve patient monitoring and ART adherence. However, perceived challenges included overburdened OTP clinic staff and limited space at the clinic to accommodate additional services. Limited privacy at the OTP clinic and its contribution to fear among HIV-positive patients of being stigmatized by their peers at the clinic was a common theme expressed particularly by patients, and often corroborated by providers. Co-dispensing ART and methadone at the clinic’s pharmacy window was viewed as a potential deterrent for patients. Providers felt that an electronic health information system would help them better monitor patients’ progress, but that this system would need to be integrated into existing health information systems. To address these potential barriers to implementing an integrated model, respondents recommended increasing OTP provider and clinic capacity, offering flexible ART dispensing options, ensuring privacy with ART dispensing, and harmonizing any new electronic health information systems with existing systems.

**Conclusions:**

An integrated methadone and ART service delivery model at the MNH OTP clinic could improve access to HIV care and treatment for OTP patients. However, specific implementation strategies must ensure that OTP providers are not overburdened and confidentiality of patients is maintained.

## Background

An estimated 500,000 people in East Africa use opioids for non-medical reasons, and on the mainland of Tanzania, there are an estimated 30,000 people who inject drugs (PWID), primarily heroin [1, 2]. The rapid escalation of injection drug use, in the context of a generalized HIV epidemic, has resulted in a high burden of HIV among PWID. HIV prevalence among PWID in Tanzania is estimated at 35% compared to 5.1% among the general population in the country [2, 3].

Though timely initiation of antiretroviral therapy (ART) is a vital component of effective HIV prevention, care and treatment, PWID are less likely to receive ART than their non-drug using counterparts [4]. PWID consistently face barriers, such as laws and policies, which limit their access to HIV prevention and treatment interventions [5]. Where programs exist, many fail to reach those who could benefit due to requirements that make it difficult for people to enter and remain in services [6]. Individual and structural barriers, such as inadequate knowledge of ART, untreated mental illness, unstable housing, fear of criminalization, and stigmatization impact the use of HIV services among PWID [7–9]. In some settings, clinicians have delayed or withheld ART from people who use drugs for fear of non-adherence and development of drug resistance [10, 11]. As a result, only 4% of HIV-positive people who inject drugs receive ART globally [4].

To address the HIV epidemic among PWID, the government of Tanzania launched the first publicly-funded opioid treatment program (OTP) on the mainland of sub-Saharan Africa in February 2011, offering daily directly observed methadone services at Muhimbili National Hospital (MNH) in Dar es Salaam [12]. At the time of data collection, in order to be eligible for enrollment into OTP, individuals had to (1) present with opioid dependence, (2) have evidence of recent drug injection, and (3) test positive for opioids through urine screening. The estimated prevalence of HIV at 39% [13] and tuberculosis (TB) at 4% [14] among PWID enrolling into the MNH OTP clinic is over 7 and 20 times [15, 16], respectively, the prevalence in the general population. A strong body of evidence supports the integration of HIV treatment into OTPs in order to improve ART initiation and adherence, and HIV viral suppression [17–19].

### Existing and proposed organization of HIV care within the MNH OTP clinic

**Fig. 1:**
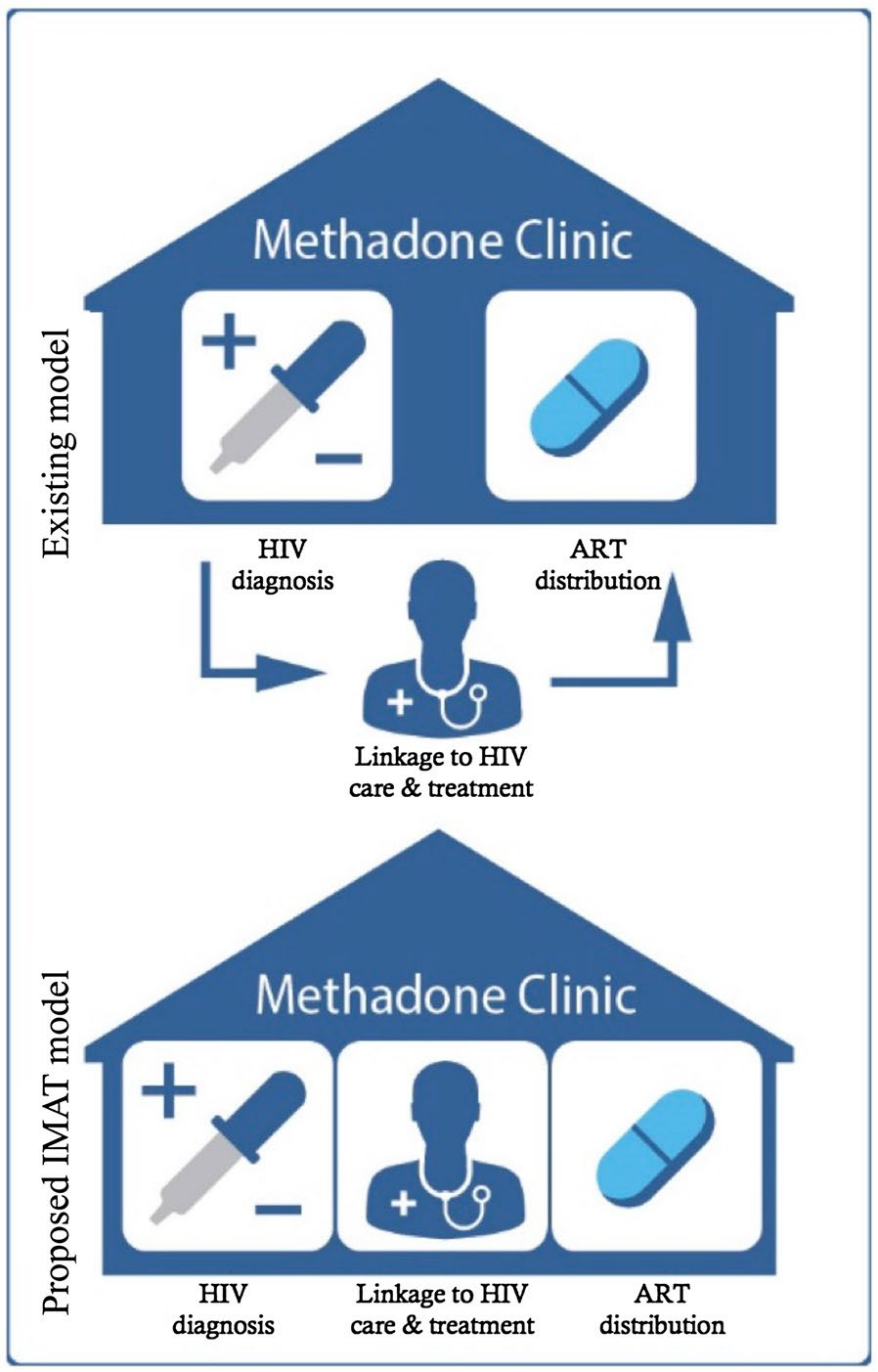
Existing and proposed organization of HIV Care within the OTP clinic

The organization of HIV care within the MNH OTP clinic can be described in three major components: (1) HIV diagnosis, (2) linkage to HIV care and treatment, and (3) ART delivery (Fig. 1). At the time of this study, HIV care was partially integrated into the OTP clinic. For HIV diagnosis, the OTP clinic offered provider-initiated HIV testing and counseling for its patients at enrollment and after every 6 months. For linkage to HIV care and treatment, HIV-positive patients could have their blood drawn at the OTP clinic, which would be sent to the central pathology laboratory for CD4 assessment. Patients were then provided an escorted referral to the HIV care and treatment clinic at MNH, located about 500 meters from the OTP clinic on the same campus, for clinical visits to discuss CD4 results, co-morbidities, other aspects of clinical management, and to initiate people on ART once they became eligible. At the time of this study, Tanzania’s national HIV management guidelines specified a CD4 count of less than 500 copies/mL to be eligible for ART [20]. Once initiated onto ART, patients could pick-up their monthly supply of ART medications from the methadone-dispensing window at the OTP clinic.

Despite daily encounters with the OTP clinic, less than half of all treatment-eligible patients at the MNH OTP clinic had initiated ART within 3 months of being deemed eligible for treatment [13]. To address delays in ART initiation and improve clinical outcomes, we proposed a more fully integrated HIV and methadone service delivery model at the MNH OTP clinic, which we called the integrated methadone and antiretroviral therapy (IMAT) model. At the time of data collection, the proposed IMAT model included four key components: (1) in-house point-of-care (POC) CD4 testing; (2) in-house HIV clinical management by OTP providers trained in comprehensive HIV management, with referrals to the HIV clinic for developing needs; (3) ART delivery through the OTP clinic; and (4) an electronic information system to help providers monitor OTP patients along the continuum of HIV care.

In this paper, we examine perceived benefits, challenges, and recommendations for the four key components of the proposed IMAT model from the perspective of patients and providers at the MNH OTP clinic as formative research, as part of a larger implementation study, to inform appropriate implementation strategies for this setting.

## Methods

In February 2015, we conducted semi-structured interviews with 12 OTP clinic providers and 20 OTP patients living with HIV at the MNH OTP clinic in Dar es Salaam, Tanzania. Providers were eligible to participate if they had worked at the methadone clinic for at least 6 months. Patients were eligible to participate if they were currently enrolled in methadone treatment at the clinic, diagnosed and on record at the clinic as being HIV-positive, at least 18 years old, and willing and able to provide informed consent. We purposively sampled MNH OTP clinic providers based on job function in order to elicit insights from providers whose jobs would be directly affected by expanding HIV care and treatment services at the clinic, which included medical doctors (6), nurses (2), pharmacists (2), and social workers (2). We purposively sampled OTP patients based on sex and ART treatment status (10 women and 10 men; 10 on ART and 10 not on ART) in order to examine differences between women and men, and also between those patients who were, at the time of the interview, currently on ART and those who had not yet started ART. Among patients sampled, the median length of time enrolled at the OTP clinic was 3 years. Among those patients on ART, the median time on ART was 1 year and 4 months.

Interview guides included open-ended questions on the perceived benefits, challenges, and recommendations on strategies to further integrate HIV care, based on the four components of the proposed IMAT model, within the MNH OTP clinic. We obtained informed consent from participants before each interview. Interviews with providers were conducted in private offices in the OTP clinic, while interviews with OTP patients were conducted in a private room in a separate building at the hospital to ensure confidentiality.

Interviews were audio-recorded, transcribed word-for-word in Swahili by the research assistant who conducted the interview, and translated into English by independent, external translators. English translated transcripts were then reviewed by the original interviewer to assess for accuracy and completeness. Throughout data collection, the study team held weekly debriefing meetings to discuss emergent themes, which helped guide interview questions to further explore in subsequent interviews for an iterative data analysis process.

We adopted a thematic content analysis approach to data analysis, framed around identifying benefits, challenges and recommendations for the four proposed components of the IMAT model. The second author (HTS) developed a codebook using a priori descriptive codes based on the interview guides and other descriptive codes that emerged from an initial coding of six transcripts. A different member of the study team involved with data collection then used this codebook to code all transcripts, adding additional codes as they emerged after discussion with the second author. This study coder and the second author then conducted a second round of coding by using the first-order, descriptive codes to develop sub-codes that further categorized interview data for analysis [21]. All coding and data management were conducted using NVivo Version 11 (QSR International, Melbourne, Australia).

This study received ethical approval from the Tanzania National Institute for Medical Research, Muhimbili University of Health and Allied Sciences, and Ethical and Independent (E&I) Review Services in the United States.

## Results

### In-house POC CD4 testing

Both patients and providers commented that the previous process of sending blood samples to the central pathology laboratory for CD4 testing resulted in delays in ART initiation for methadone patients. An integrated model that includes POC CD4 testing and ART initiation at the MNH OTP clinic would eliminate delays resulting from off-site CD4 testing:

[Having CD4 testing capabilities at the methadone clinic]…would help us to control the timing of CD4 testing. You may find a client comes to you and you realize that he needs to undergo a CD4 test because he already has opportunistic diseases. But currently you have to draw blood and request to have him tested [at the central pathology laboratory]. You find you are wasting time waiting for the results. (Nurse)

Though POC CD4 testing at the OTP clinic was widely reported among providers, in particular, as an opportunity to reduce delays in ART initiation, a few providers highlighted limited trained OTP personnel and space available at the clinic to house the machine as potential barriers to implementation and recommended increasing the number of OTP personnel trained in administering the CD4 tests and the clinic capacity to accommodate any new services. As one doctor at the OTP clinic explained:

[H]uman resources are very important to increase so that the services are maintained as they were before. Because you can’t ask a person who was dressing [wounds] to dress [wounds] and at the same time to [administer a] CD4 [test], you see? But if there is another person allocated for that activity, it would be good. (Doctor)

### In-house HIV clinical management

Integrating HIV clinical management at the OTP clinic, to create a “one-stop shop,” was perceived by OTP patients as improving access to HIV care and treatment services, by making these services more convenient for patients. Mostly patients, though a few providers as well, reported having to seek HIV care and treatment at off-site HIV clinics as a deterrent for many OTP patients from initiating and adhering to ART medication, partly due to overcrowding. As one patient described:

Someone might go [to the HIV clinic] for treatment and find a long line with many people. He might think, “I’m late for my personal activities. I’ll come back tomorrow.” Tomorrow again he comes and finds the same long line: “Ah, let me come back tomorrow.” And when you stay three days without taking ARTs, I heard there is a problem because every day you have to get your medicine. So [an integrated methadone and ART model] would help in some way. (Female patient on ART)

Another patient elaborated further:

At the HIV clinic, I don’t know, most [methadone patients] when they are told to go and get medication, they say there is a long queue with a lot of disturbances. But I think that if [HIV treatment] services are brought here, most of [patients] would make the sacrifice themselves and decide to take [ARTs]. It would be faster. (Female patient on ART)

Stigma due to a history of opioid use and addiction was also reported by patients and providers as a deterrent to HIV care-seeking among OTP patients. Many patients and providers expressed that an integrated model of care would reduce OTP patients’ exposure to stigma and discrimination when seeking HIV care and treatment at off-site HIV clinics, and ultimately increase their access to these services. Patients and providers hinted that the stigma encountered by OTP patients at outside HIV clinics was not just from other HIV-positive patients seeking care, but also HIV clinic providers. As one provider explained:

I am among those who fought for ART and methadone to be offered simultaneously [at the OTP clinic]. The first reason, I observed high rates of stigma when we started to offer methadone. We didn’t have ART in our clinic; we did not have it in our pharmacy. What was happening was after we diagnosed [someone with] HIV and if that person was eligible to start ART, we referred that person to the [off-site] HIV clinic… For [patients] this was a big problem… They were being labeled and stigmatized. There was offensive and stigmatizing language that made many of them discontinue [HIV care and treatment]. And in the beginning, most did not use ART for that reason. (Senior doctor)

Many clinical personnel at the OTP clinic are trained mental health specialists, with primary appointments in the hospital’s psychiatry and mental health ward. They have experience caring for patients with histories of opioid use and behavioral disorders. Many OTP patients expressed positive feelings toward the care they receive from providers at the OTP clinic, and viewed this as an advantage to receiving additional HIV clinical services as part of the proposed IMAT model at the OTP clinic:

I: [W]hat would be the advantages of getting HIV services from [the OTP clinic] rather than going somewhere else in the hospital or another hospital?

R: I mean, like here [at the OTP clinic] we are family. There is nothing like discrimination or humiliation. So it would be a relief for us because [the OTP providers] know us. (Female patient on ART)

Providers perceived that an integrated model at the OTP clinic could improve access and adherence to HIV treatment:

Many of our clients would agree to receive those HIV services because right now the majority refuses because HIV services are rendered from another clinic different from ours here. So I think many more would agree to start ARTs and they would agree to get those services because they are offered from one place. (Senior doctor)

According to providers, offering expanded HIV treatment services at the OTP clinic would not only make it easier for patients to initiate and remain on ARTs, but would also allow providers to better move patients along the HIV continuum of care by being more involved in and aware of their care.

The opportunities to increase access to HIV care and treatment among OTP patients that would be afforded by more fully integrating HIV clinical care at the OTP clinic were often expressed by providers alongside the need to adequately train OTP providers in the clinical management of HIV-positive patients and to take into consideration the current high volume of patients seen on a daily basis at the OTP clinic.

### ART delivery through the OTP clinic

In the proposed IMAT model, ARTs would be co-dispensed at the pharmacy window where methadone is currently dispensed. Patients, especially, but providers as well, were concerned that the lack of privacy at the OTP clinic window (where patients take their methadone) could result in unintended HIV status disclosures to their fellow patients since the pharmacy windows at the OTP clinic are open to the waiting area:

I think this service needs to continue being confidential because most of them stigmatize. So there if you dispense from the window, it is when people [other OTP patients] will see that someone has what, has AIDS. And you should know that the patient’s secret is between himself and the doctor. Now there you reveal it out for everyone to know. (Male patient not on ART)

The disclosure of one’s HIV status through a lack of privacy at the pharmacy window could result in stigma and discrimination by peers, as one patient explained:

It’s not good because when someone finds out that you are infected, know that fingers will be pointed towards you. “Ah there he goes. He is taking medicine! There he goes!” Because now I am infected. Someone has only a chest problem and they talk about him. What about me who is infected [with HIV]? And again there is the issue of love. We fall in love among ourselves. So you see that if my lover sees me taking pills there at the window, that container, she would stare at me. She wouldn’t love me anymore. She would run away from me. You see? For that small thing, stigma would persist. (Male patient on ARTs)

As a way of addressing the privacy issues at the clinic’s pharmacy window, patients echoed recommendations also made by providers to dispense ARTs in a separate, more private location at the OTP clinic to mitigate HIV-related stigma. When asked how to maintain confidentiality when offering ART at the clinic, one patient responded:

If someone knows your problem, it becomes a caning stick to you. Another window is required so that other people won’t know what you are going to do or even to know that you are going there. You would just know yourself. (Female patient on ART)

Patients also reported ART medication adherence as a potential challenge to co-dispensing ART medications and methadone in light of current clinic policies regarding late arrivals. Methadone is dispensed at the OTP clinic daily from 6 a.m. to 11 a.m. If a patient arrives after clinic dispensing hours, according to clinic policies, she or he is denied his or her methadone dose: “If you come late, they do not receive you. Which means if you miss a methadone dose, then you would have to miss an ART dose [as well].” (Male client not on ART) This was viewed as particularly problematic given the gravity of non-adherence to ART medication.

Patients, especially, expressed that if the proposed IMAT model is implemented at the OTP clinic, then there should be flexibility in clinic policies regarding ART dosing and dispensing. Methadone is administered once daily in the morning at the OTP clinic, however some patients reported a preference to take ART medications in the evening, in the privacy of their homes, or at a time they might be more likely to have food. An option for take-home dosing of ART medications—such as a monthly supply of medication as practiced at HIV clinics—rather than daily, observed therapy was described by patients as another option for ART dosing and dispensing to ensure privacy. One patient reported his preference for take-home dosing:

For me it is better because I take my [ART] pills [home]. I drink my [methadone], I take my pills and leave with them. Because I cannot take my pills [at the OTP clinic]. (Male patient on ART)

Lack of privacy at the OTP clinic and its contribution to fear among HIV-positive patients of being stigmatized by their peers at the clinic because of their HIV status was a common theme expressed particularly by patients interviewed, and often corroborated by providers.

There is no privacy for patients, so it becomes a problem. So if people see him coming to the window for [ART] medicines, then it would be a problem. So there is the possibility that some may stop [coming to the OTP clinic] due to stigma. And so there is a need for privacy. (Pharmacist)

Co-dispensing ART medications with methadone as part of the proposed IMAT model at the OTP clinic was also perceived by OTP providers as adding more work for already overburdened OTP clinic personnel, and might, consequently, lead to delays in ART, as well as methadone, delivery at the clinic.

To dispense methadone, I have to observe his behavior: has he taken alcohol; has he smoked weed; does he have a behavior that is not appropriate. It is very important that he is observed so that he can be assisted. Now when I am through observing him, I give him methadone. It takes like three minutes to do those observations, but later on I would need to give him ART medicine. He would need to go around to the window so that I could give him his medicine. So it may start to delay the rendering of services. (Pharmacist)

### Electronic health information system to track OTP patients along the HIV care continuum

One of the proposed components of the IMAT model at the OTP clinic was an electronic health information system that would serve as a platform to: (1) store patients’ HIV-related data, such as HIV testing, ART testing, and treatment results and status updates; (2) send out alerts to providers on any required testing needed; and (3) remind providers of the next steps to take to move patients along the continuum. Most providers reported that this type of electronic system would help them to better monitor patients’ progress along the HIV continuum of care in real time and more effectively manage their HIV care, compared to the existing paper-based system, by improving their ability to quickly retrieve and access important patient HIV-related data to make clinical decisions. Yet providers stressed that OTP clinic’s electronic health information system for managing HIV-positive patients would need to be harmonized with the existing methadone treatment data available at the clinic.

But if they could find a way to harmonize the information about HIV/AIDS [with the existing methadone data] it would be easy because the information would flow in the same manner. So when I open it, I would know now he is at a certain dosage of methadone, this one’s CD4 information was taken on this date, okay, he has already started ART and is doing fine and he is due for another CD4 test… in general, to see progress and treatment failure. (Doctor)

Providers also recommended ensuring that any electronic HIV-related alert-and-reminder health information system adopted by the OTP clinic be interoperable with existing laboratory and national HIV electronic health information systems currently used at the hospital.

Providers perceived barriers to adopting an electronic health information system at the OTP clinic to manage the HIV care of patients that would need to be addressed when developing specific implementation strategies and protocols for the IMAT model. Electrical power outages were reported as sporadically occurring at the OTP clinic, which could affect the ability of OTP providers responsible for the management of HIV care of patients to access the information necessary to make clinical decisions. Problems with the computer server being down could also limit timely access to patient data. Providers revealed how the computer server that provided data sharing capabilities to the OTP clinic for managing patient’s methadone treatment was non-functional for over a year. As a result, OTP providers had to revert back to using paper-based forms, which made retrieving and accessing patient data difficult and time-consuming. In light of this, some providers recommended developing plans and protocols for maintaining and repairing the electronic health information system and its associated equipment, when needed:

There should be a good system that when it is broken, maybe there should be a technician or someone who will be close to take care of it, so that when they are broken, they can be fixed and the service continues instantly, and not that the service should be halted for a long time. (Senior Doctor)

In concurrence with the cross-cutting theme around issues of privacy and confidentiality with fully integrating HIV services at the OTP clinic, to maintain patient-provider confidentiality a provider also recommended that security access certificates be provided to OTP providers based on the patient information required to carry out their assigned clinic duties.

## Discussion

This study described the perceived benefits, challenges and recommendations for implementation regarding the integration of HIV care into the OTP clinic at Muhimbili National Hospital. Our findings highlight the importance of integrating HIV care into the OTP clinic setting in order to reduce stigma and discrimination and leverage the compassionate care offered by OTP clinic providers. However, specific attention will be needed to structure services so that providers are not overburdened and confidentiality regarding patients’ HIV status is maintained.

At the time of data collection there were 17 clinicians, 6 postgraduate residents (that serve on a rotating basis) along with 3 nurses, 4 pharmacists and 3 social workers that served the OTP clinic. While the addition of HIV care and treatment would add to provider work responsibilities, the addition of these services alone would not increase the number of patients enrolled at the OTP clinic. The combination of OTP and HIV services would help to address patient concerns around the time required to receive both kinds of care. However, providers recognized the clinic level changes that would be needed to make these changes. Providers also felt that for integration to be successful, they would need a system to manage and monitor patient care, which would be fully functional and include technical assistance. Findings from this study have been used to inform the development of a model to integrate HIV care into OTP in Dar es Salaam, Tanzania.

The management of HIV among people who use drugs is key to addressing the intersecting epidemics of HIV and drug use [4]. This is especially important as existing research has demonstrated the spread of injection drug use in East Africa, along with a high burden of HIV [13, 22]. It is, therefore, critical to develop HIV care models that will facilitate utilization of HIV services for this key population. Engaging OTP patients who would access and providers who would implement HIV care and treatment is critical to build delivery approaches that factor in culture and context and respond to the needs of communities that interact with them [23]. The ability of OTP providers to care for patients is especially relevant as other (non-OTP) clinicians may be resistant to working with PWID. This creates an environment at the OTP clinic conducive to compassionate care based on respect for patients and that acknowledges their unique vulnerabilities and struggles.

Previous research conducted at the MNH OTP clinic has identified challenges around initiating HIV-positive OTP patients onto ART, including delays in CD4 testing, the inconvenience of off-site HIV clinics, and stigma [12]. The IMAT model was refined through this research to address many of the challenges identified with the intention of improving the provision of HIV care for OTP patients. OTP providers (clinicians and nurses) have medical expertise that can be enhanced with additional training to provide HIV care and treatment. HIV-positive OTP clients are familiar with, and comfortable in, the OTP clinic environment where they feel respected by OTP providers. For these reasons, the IMAT model is, potentially, a more feasible intervention than providing OTP in existing HIV clinics.

Patients and providers were broadly supportive of integrating HIV care into the OTP clinic. As a health service model, OTPs can facilitate the improvement of health-related outcomes for their patients and have proven to be a successful venue for treatment of infectious diseases such as HIV [24–27]. Additionally, research shows that combining OTPs, such as methadone maintenance, and ART is both cost effective and helps care providers to monitor and observe patient treatment [28]. OTP clinics in Tanzania provide a unique opportunity for comprehensive HIV care to this high-risk population for several reasons. First, the HIV burden among PWID is 7-fold greater than what is observed in the general population [13]. Second, methadone helps to stabilize PWID and can facilitate adherence and completion of treatment regimens [29, 30]. Third, methadone treatment decreases injecting behaviors and therefore decreases transmission and the potential for reinfection. Fourth, OTP providers are perfectly positioned to provide culturally competent services for PWID, compared to most other health care providers, and can assist in the cultivation of a welcoming environment for the provision of services. Fifth, as the majority of patients present daily for their methadone dose, OTP clinics are ideal venues to administer once-daily medication, monitor treatment, and provide proper follow-up care, including risk prevention counseling.

Researchers have engaged communities of interests in the interpretation of findings, to translate research findings into implementation strategies [31, 32]. Key stakeholders, including patients, community outreach workers, providers, and policymakers, played a key role in interpreting this study’s findings and shaping the final design of the IMAT service delivery model through community and partner engagement meetings. For example, as part of the proposed IMAT strategy we originally planned to train two dedicated HIV care and treatment specialists within the OTP clinic. However, in light of feedback provided by key stakeholders during the engagement meetings, we realized that the original approach might be too limiting and that it would be more beneficial to build HIV care and treatment capabilities among more clinicians, including both physicians and nurses, within the OTP clinic to prevent increasing the workload and overburdening individual providers. Training several clinicians would also provide flexibility in scheduling patients for clinical visits and follow-up consultations, increasing the capacity of the MNH OTP clinic to provide HIV care to patients.

Perhaps the greatest concern of the patients and providers interviewed for the study was maintaining confidentiality regarding their HIV status. This concern was echoed during the community and partner engagement meetings. OTP patients concerns around HIV serostatus disclosure should be especially respected in terms of non-discrimination related to HIV care and treatment; however, this can be a problem in the context of non-disclosure to sex partners. Fear of inadvertent disclosure by accessing HIV care within an integrated setting has been previously reported in the literature [33]. To address this concern, the revised IMAT strategy allows patients to select one of three ART dispensing models: (1) directly administered ART by a clinician in a private setting, (2) directly administered ART at the methadone dispensing window, or (3) monthly supplies of ART, which is the standard of care in Tanzania.

This study has limitations. We only interviewed patients currently enrolled and providers working at the MNH OTP clinic. Patients who have defaulted or who were not currently enrolled in care for other reasons may have very different perspectives around these issues. The MNH OTP clinic is one of four OTP clinics in Tanzania. Findings from this study and the integrated HIV and methadone delivery model that was developed to address some of the challenges and recommendations identified by participants, may not be applicable to the other OTP clinics given the high-level of resources available at the MNH OTP clinic.

## Conclusions

Building on our previous implementation science research [12, 13, 23, 33–38], this study sought examine how to best integrate HIV services into the MNH OTP clinic from the perspectives of OTP patients and providers. Our findings suggest broad support from patients and providers regarding integrating HIV care within OTP, but highlight that implementation strategies must ensure that the confidentiality of patients is maintained with an integrated model. As this study examined perceptions regarding a proposed integrated care model, future implementation research will focus on understanding the implementation of the integrated model and its impact on improved access to HIV services for OTP patients.

## Abbreviations

PWID: people who inject drugs
ART: antiretroviral therapy
OPT: opioid treatment program
MNH: Muhimbili National Hospital
TB: tuberculosis
IMAT: integrated methadone and anti-retroviral therapy initiative
POC: point-of-care
